# Smart home system using blockchain technology in green lighting environment in rural areas

**DOI:** 10.1016/j.heliyon.2024.e26620

**Published:** 2024-02-17

**Authors:** Ying Huang

**Affiliations:** College of Art & Design, Putian University, Fujian, China

**Keywords:** Access control, Blockchain technology, Control model, Green lighting, Smart home

## Abstract

Currently, with the rapid development of smart home technology, the demand for establishing efficient and sustainable smart home systems in rural areas is increasing. However, in rural environments, the effective management and intelligent control of green energy face many challenges. To address these issues, this work aims to design a smart home system based on blockchain technology to achieve efficient energy management and intelligent control in a green lighting environment in rural areas. The main goals include improving the performance and safety of the system to meet the lighting needs of rural areas and promote sustainable development. The system comprises two primary components: the home gateway and cloud services. These components encompass functions like data monitoring and transmission, cloud storage, and remote control. The work also introduces the structural interaction, user node interaction, and the data security transmission scheme of the smart home system. Ultimately, the system's effectiveness is confirmed through simulation experiments. The results demonstrate that the system achieves the lowest latency when the transaction arrival rate is 40tps and the block size is 10. Additionally, the access control scheme based on the Hyperledger Fabric consortium chain can efficiently handle access requests for smart home resources and meet the practical application requirements within an appropriate range of security parameters. The main research conclusion is that the designed smart home system based on blockchain technology has achieved significant results in improving performance and security. This not only provides reliable lighting solutions for rural areas, but also provides important theoretical and practical guidance for the future development of smart home systems. The direction of future work includes further optimizing system performance, expanding the scope of application, and exploring more advanced blockchain technology applications in the field of smart homes. This will provide more possibilities and innovative directions for the development of future smart home systems.

## 1 INTRODUCTION

### 1.1 Research background and motivations

In the era of 5G and big data, the concept of “Internet +" is sweeping all industries. In this context, the "+" refers to the fusion of traditional industries with information technology through the internet. This integration has given rise to new industrial forms and business models while driving various industries' digitization, intelligence, and informatization. Supported by national policies and advancements in science and technology, a series of lifestyles such as “wisdom +" have developed rapidly [1]. Many intelligent fields have mentioned “intelligent manufacturing", which fully demonstrates the government's support for the “intelligent" industry. Smart homes have emerged as a prime example of this technological shift, where users can control household appliances and devices via networks or transmission protocols [2]. The advent of smart appliances has linked people's lives with the internet, reshaping work environments and lifestyles. The rapid development of smart home technology has provided new possibilities for improving quality of life and increasing energy efficiency. With the rapid advancement of science and technology, artificial intelligence, machine learning, and data analysis are crucial foundations for developing science and technology. While accelerating mankind's entry into the era of the Internet of things (IoT), the development of information technology has greatly facilitated people's lifestyle. It allows increasingly busy individuals to free themselves from complex household chores, making their lives safer, more comfortable, convenient, and efficient. Sepagozar et al. (2020) fully understood the gaps in the application of intelligent building environments based on the IoT, and proposed methods for designing smart homes that enabled users to save energy and reduce greenhouse gas emissions while improving thermal comfort [3]. Machorro-Cano et al. (2020) pointed out that the progress in energy conversion, as well as the emergence of new communication and information technologies, had paved the way for the development of smart homes. The IoT, as a fusion of various heterogeneous technologies, connects various devices through the internet to achieve detection, monitoring, and remote control of multiple devices [4]. Especially in rural areas, achieving efficient energy management and intelligent control is crucial for promoting sustainable development. Zhang et al. (2020) focused on the challenges facing sustainability in rural areas of China and the Chinese government's decision to build smart villages as an important strategy for achieving sustainable rural development. The research results indicated that the role of the Chinese government in promoting the construction of smart villages was mainly reflected in promoting the strategic subsystems of smart village systems through unified overall planning and relevant support policies, thus enhancing the development of smart rural areas [5]. Saleem et al. (2023) proposed an intelligent energy management system for intelligent environments, which achieved efficient demand side management by integrating energy controllers and IoT middleware modules [6]. However, traditional smart home systems often face many challenges in the face of increasing energy demand and environmental protection pressures. Mallinson et al. (2022) argued that smart home technology was a means to improve quality of life and independence, while reducing the long-term cost of caring for the elderly population. However, SHT has also brought many governance and policy challenges, especially in the protection and use of massive patient data [7]. Haque et al. (2022) believed that the pinnacle of the development of smart cities was the innovative integration of the latest information and communication technologies. Citizens of smart cities can enjoy intelligent living environments, ubiquitous connectivity, seamless access to services, intelligent decision-making through intelligent governance, and optimized resource management. The widespread acceptance of smart cities has led to a series of issues such as data security, identity verification, unauthorized access, device level vulnerability, and sustainability [8].

With the booming development of the smart home IoT industry, security issues, especially information security, have gained prominence, drawing the attention of smart home users. Currently, the security strategies in smart homes primarily rely on traditional centralized approaches, which encounter challenges such as high system overhead and weak scalability in security management [9]. In 2009, blockchain technology (BT) emerged as a game-changer. It boasts features like decentralization and non-tampering, making it highly effective in establishing trust mechanisms in unfamiliar environments. BT excels in security and has found applications in various industries, including digital currency, where it ensures the security and credibility of transactions through decentralized records. Furthermore, BT can also be applied in areas such as supply chain management, electronic contracts, and intellectual property protection to enhance transaction transparency, traceability, and security. It offers a promising solution to network security challenges in IoT applications with the smart home as a typical scenario. BT can address network security problems and challenges in IoT applications with the smart home as a typical scenario. This work explores the utilization of BT to address access control and network security issues in smart home scenarios through a combined analysis of BT and IoT technology application scenarios [10].

In the current research area, BT has garnered widespread attention as a critical pillar of security for smart home systems. Existing BT models comprise public blockchains, represented by Bitcoin, and consortium chains, typified by Hyperledger Fabric. Public blockchains offer decentralization and tamper resistance but still face scalability and energy consumption constraints. Consortium chains address some of public chains' scalability and privacy concerns but come with challenges related to centralized management and a lack of trust. Many scholars have conducted thorough discussions and comparisons of these BT models within the research field. Mansouri et al. (2021) highlighted the decentralization characteristic of public blockchains, which ensured data security and trustworthiness. However, due to their primary design for supporting cryptocurrency transactions, public blockchains may encounter performance bottlenecks and high energy consumption in practical applications [11]. In contrast, Zang et al. (2019) emphasized the advantages of consortium chains in privacy protection and enhanced transaction efficiency. They also highlighted the higher trust requirements among participants and certain scalability challenges associated with consortium chains [12].

In modern society, smart home systems have been widely applied in urban areas. However, in rural regions, especially in the context of green lighting, significant room exists for development. This work aims to explore the application of BT-based smart home systems in rural areas with green lighting. Existing literature and practices lack related comprehensive research on this. Thus, this work intends to bridge the existing research gap and explores a smart home system based on public blockchain technology, aiming to achieve more efficient energy management and smarter control in green lighting environments in rural areas. The distributed, secure, and tamper proof features of blockchain technology have brought new solutions to the field of smart homes, which are expected to improve system performance and reliability (Nakamoto, 2008; Swan, 2015). In terms of research scope, this work includes two main components: home gateway and cloud services. These two jointly build a system that realizes core functions such as data monitoring and transmission, cloud storage, and remote control (Li et al., 2022). By combining these components, this work proposes a feasible system architecture to meet the lighting needs of rural areas and improve energy efficiency. The main contributions of this work include the introduction of structural interaction, user node interaction, and data security transmission schemes. The effectiveness of the system is verified through simulation experiments, and the results show that the minimum delay can be achieved under specific parameters. In addition, an access control scheme based on the Hyperledger Fabric consortium chain has also been introduced to ensure secure access to smart home resources. This work expects to provide strong support for the application of smart home systems in rural areas and provide useful theoretical and practical experience for future research and development.

### 1.2 Research objectives

The research motivation stems from the numerous challenges that smart home systems face in green lighting development in rural areas. In this context, BT has garnered significant attention as an emerging means of ensuring security. However, existing literature lacks research on blockchain applications for intelligent management of green lighting in rural areas. Hence, this work aims to design a BT-based smart home system to achieve efficient energy management and intelligent control in rural green lighting environments. This system encompasses functions like data monitoring and transmission, cloud storage, end-user displays, and secure data uploading based on blockchain technology. Additionally, this work explores the application of BT-based smart appliances in rural green lighting to provide a secure and reliable smart home platform. The achievement of this objective can significantly advance the level of intelligence in rural green lighting, offering fresh solutions to the challenges of traditional rural lighting energy management and intelligent control.

The innovative contribution lies in the integration of BT with smart home systems, providing a novel intelligent management model for rural green lighting. Through the application of BT, this work ensures the secure transmission and storage of data and enables efficient control and management of smart appliances. Given the lack of comprehensive research in existing literature and practice on BT-based smart home systems in the context of rural green lighting, this work fills a significant research gap and offers new theoretical and practical guidance for intelligent lighting management in rural areas. This innovative contribution can drive the development of smart home systems in rural areas, particularly in the field of green and energy-efficient lighting, thus bridging the research gap in existing literature and practice.

## 2 Literature review

### 2.1 Security technology for smart home

On this basis, Dang proposed a data security technology for smart homes based on BT. This architecture was illustrated through Ganache, Remix, and web 3. The smart contract in SHIB was evaluated based on users, service providers and smart homes [13]. Experiments demonstrated that the SHIB system exhibited the characteristics of data privacy, trust access control and good scalability. Furthermore, the proposed architecture was compared with existing models, considering factors like smart contracts, data privacy, token usage, policy updates, and wrong behavior judgments [14]. Tchagna proposed a blockchain method to protect data in the IoT architecture. Blockchain was adopted for its scalability, flexibility and improved native performance. The architecture for implementing blockchain in smart homes was also introduced. Arduino, Raspberry Pi, and sensors were applied to establish an IoT ecosystem for smart homes [15]. The privacy, integrity and accessibility of the blockchain-based smart home architecture were carefully evaluated. Additionally, the simulation results were presented to emphasize that this method's additional costs (distribution, processing time and energy consumption) were unrelated to the system's protection and privacy [16]. The smart home automation system has been increasingly used in IoT since its emergence. Wireless networks are adopted to apply sensors to household appliances, enabling residents to control these devices remotely. However, storage and processing capacity limitations have led to substantial security risks, rendering these devices susceptible to a range of attacks. Consequently, ensuring security has become an essential research focus for deploying these devices and has gained significant attention from researchers. Furthermore, traditional security strategies are inadequate in effectively addressing the security issues associated with these devices. As a distributed database based on encryption technology, many scholars have been dedicated to studying the role of blockchain in ensuring the intelligence and security of IoT [17]. A thorough investigation into the application of BT in households is conducted. A blockchain-based approach that uses authentication certificates to manage electronic devices in smart homes is proposed, implemented and evaluated. Furthermore, this system is compared with the traditional system based on work verification [18]. Ren et al. (2021) employed incremental aggregated sub-vector commitments to replace the Merkle trees to store data in industrial IoT. Storing individual commitments rather than the entire qualification list helped alleviate the storage burden on nodes [19].

### 2.2 Application of blockchain in smart home networks

Ammi et al. (2021) introduced a novel secure smart home system solution based on blockchain, utilizing a combination of Hyperledger structures and a Hyperledger editor. Another crucial aspect of the proposed solution was mapping smart home attributes to the Hyperledger editor's attributes [20]. Farooq et al. (2022) proposed a smart home network architecture based on a private blockchain for intrusion detection. They delved more extensively into the smart home network framework's various key components and functions [21]. Menon et al. (2023) presented a learning engine for smart home communication networks, which leveraged blockchain-based secure communication and cloud-based data evaluation layers. This engine separated and ordered data based on three categories of transactions: Smart T, Mod T, and Avoid T. This ensured a secure and efficient smart home communication network [22].

### 2.3 Network management security of blockchain and edge computing

Liao et al. (2021) focused on two key components of security and forensics in network management, particularly to ensure reliable operations for large-scale access networks such as the IoT [23]. Blockchain, as a decentralized shared ledger and database, is considered to provide collaborative trust and collaborative action among multiple entities while ensuring data integrity and confidentiality. Due to its anonymity, tamper resistance, and traceability, blockchain has triggered research on device security, data security, and forensics in the IoT in combination with blockchain and edge computing. This work analyzes the application of blockchain in mobile edge computing IoT system, focusing on the methods and technologies to solve the security and forensics problems of IoT.

### 2.4 Efficient verification of blockchain in industrial IOT

Wang et al. (2021) proposed an optimized Merkle tree structure to achieve efficient transaction verification in trusted blockchain enabled industrial IoT systems. The new Merkel tree structure is more effective in verifying blockchain transactions in trusted blockchain-enabled industrial IoT systems [24]. Dhanaraj et al. (2022) proposed a novel Probit Regression Davis Mayer Kupyna Cryptographic Hash Blockchain (PRDMKCHB) technique, which used Kupyna cryptography to generate hash values for each data [25]. The Davis Mayer compression function is used to improve the security of data transmission and reduce packet loss. Conduct comprehensive simulations to verify the performance of the proposed PRDMKCHB technology and existing blockchain technologies in terms of data transfer ratio, data loss rate, and execution time. The simulation results showed that compared with previous blockchain technologies, the proposed PRDMKCHB technology had better performance in packet transfer ratio, minimum packet loss, and execution time. Zhang et al. (2020) summarized existing blockchain-based systems and applications. They mainly reviewed the application of blockchain traceability technology in various fields, decentralized application of blockchain, and other blockchain applications in data security protection, bringing new opportunities and challenges for the development of various industries in the future [26]. Wang et al. (2020) utilized a password accumulator instead of a Merkle hash tree to provide member proof and non-member proof. They proposed a new type of unbounded accumulator and provided its definition and security model [27]. Finally, they constructed an unbounded accumulator scheme using bilinear pairs and analyzes its performance.

### 2.5 INTELLIGENT manufacturing scheme for deep learning and blockchain

Singh et al. (2021) proposed a scheme called DeepBlockScheme, which combined blockchain and deep learning to ensure the integrity, decentralization, and security of manufacturing data. In the proposed scheme, blockchain was distributed and deployed in the fog computing layer to ensure the integrity and security of manufacturing data. Deep learning was used in the cloud computing layer to improve production efficiency, automate data analysis, and increase communication bandwidth for intelligent factories and manufacturing applications [28]. Ren et al. (2021) proposed an identity-based proxy aggregation signature scheme to improve the efficiency of signature verification, while compressing storage space and reducing communication bandwidth. The experiment proved that, although the communication cost of this scheme was only 12%–39% of that of a regular signature scheme, its storage performance in the blockchain was improved by 20% compared to the blockchain itself [29]. Ren et al. (2019) proposed a mechanism combining blockchain and regenerative coding to improve the security and reliability of stored data under edge computing [30].

### 2.6 Summary

Table 1 shows previous research findings by scholars.

**Table 1:** previous research findings.

Scholars	Year	Research topics	Method/Technology	Results/Findings
Dang	2018	Data security technology	BTbased smart home	The SHIB system demonstrates data privacy, trust access control, and good scalability. Compared with existing models, factors such as smart contracts, data privacy, token usage, policy updates, and misbehavior judgment were considered.
Tchagna	2022	Blockchain protected IoT data	Smart home architecture using blockchain	A careful evaluation of the privacy, integrity, and accessibility of blockchain based smart home architecture was conducted, and simulation results emphasized that the additional cost of this method was independent of system protection and privacy.
Ren et al.	2021	Industrial IoT data storage	Incremental aggregation subvector commitment	Incremental aggregation of sub vector commitments was used instead of Merkle trees to store data, which reduced the storage burden on nodes.
Ammi et al.	2021	Blockchain smart home system	The combination of Hyperledger structure and Hyperledger editor	A blockchain based smart home system solution was proposed by mapping smart home attributes to the attributes of the Hyperledger editor.
Farooq et al.	2022	Smart home network architecture	Intrusion detection based on private blockchain	In-depth research was conducted on the key components and functions of the smart home network framework.
Menon et al.	2023	Smart home communication network	Blockchain-based learning engine	Blockchain-based secure communication and cloud-based data evaluation layers were utilized to ensure a secure and efficient smart home communication network.
Liao et al.	2021	Blockchain and edge computing	Application of mobile edge computing-IoT system	The combination of blockchain and edge computing in the IoT was discussed to solve the problems of device security, data security, and forensics.
Wang et al.	2021	Efficient transaction verification for industrial IoT	Optimized Merkle tree structure	An optimized Merkle tree structure was proposed to achieve efficient transaction verification in a trusted blockchain enabled industrial IoT system.
Dhanaraj et al.	2022	Probit Regressive Davis Mayer Kupyna Cryptographic Hash Blockchain	Blockchain and Kupyna cryptography	A novel blockchain technology was proposed to generate hash values for each data using Kupyna cryptography.
Zhang et al.	2020	Blockchain-based systems and applications	The application of blockchain traceability technology	Blockchain-based systems and applications were summarized, with a particular focus on the application of blockchain traceability technology in various fields.
Singh et al.	2021	DeepBlockScheme	The combination of blockchain and deep learning	A solution combining blockchain and deep learning was proposed to ensure the integrity, decentralization, and security of manufacturing data.
Ren et al.	2019	Data storage and connection of smart homes	Identity-based proxy aggregation signature scheme	Data storage security was strengthened by introducing blockchain technology.
Ren et al.	2021	The Importance of edge computing in intelligent computing	The combination of blockchain and regenerative encoding	The data storage advantages of edge network devices and cloud storage servers were utilized to build a global blockchain at the cloud service layer, while also building a local blockchain at IoT terminals. In addition, regenerative encoding wad applied to further improve the reliability of data storage in blockchain. Finally, a mechanism for regularly verifying data hash values in global blockchains was proposed to ensure the integrity of the data stored within it.

In conclusion, relevant scholars are dedicated to addressing security and privacy issues in smart homes using BT technology. Significant contributions have been made in the field of smart home security, proposing various solutions based on blockchain and other technologies to address security and privacy issues in smart home networks. By introducing technologies such as blockchain and deep learning, new architectures and methods are designed to effectively ensure the data privacy, trusted access control, and scalability of smart home systems. However, these studies have some shortcomings, such as the potential lack of comprehensive systematic performance evaluations or limitations in implementing specific security measures. Compared to previous research, this work primarily fixes the algorithm for the loopholes in the current research. It combines BT with the current smart home access control technology to access trusted BT entities. Additionally, this work integrates attribute-based access control models with BT to construct a new smart home system in the context of green lighting environments in rural areas. It exhibits uniqueness in adopting a consortium blockchain structure based on Hyperledger Fabric. It strongly emphasizes real-time performance, blockchain security, and system scalability within the context of smart home access control systems. The research methodology underscores effective control over the delay in accessing smart home resources and provides a comprehensive performance analysis of blockchain-based smart home access control systems. It highlights that the proposed solution enhances system security while maintaining high processing efficiency. This work addresses a research gap in performance evaluation that may exist in this field, providing valuable insights for advancing the smart home domain.

## 3 Design of smart home system based on BT

Holistic integration of general and scene lighting facilitates the symbiotic development of people and the environment, heralding a new era of lighting applications centered around integration, intelligence, and energy saving [31].

However, current smart home systems confront data security and privacy protection issues In order to address this situation, numerous studies have adopted BT. Unlike traditional centralized systems, blockchain offers a distributed and decentralized approach that enhances data security and provides tamper-resistant performance. Additionally, BT can offer better privacy protection mechanisms by incorporating features such as smart contracts and anonymous identity verification to safeguard user privacy. Many studies have demonstrated blockchain's effectiveness in addressing data security and privacy protection concerns within smart homes. This work utilizes blockchain perceptual computing technology. Based on the Representational State Transfer (REST) technology of network service systems, various intelligent devices are defined using Uniform Resource Locator (URL) technology for data storage [32]. However, achieving interaction between individuals and devices and between devices themselves necessitates the application of third-party APIs to ensure architectural scalability. Therefore, the system design presented adopts a distributed and extensible architecture rooted in the REST style.

**Fig. 1:**
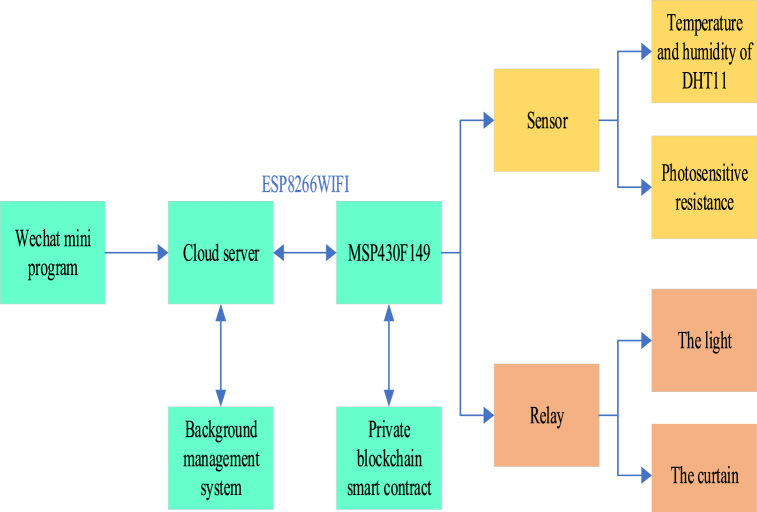
Framework diagram of the smart home system based on BT.

On this basis, this work proposes a smart home system based on blockchain, as illustrated in Fig. 1.

Fig. 1 illustrates that the BT-based smart home system consists primarily of the home gateway and the cloud server. The home gateway mainly includes the sensor module, relay module, MSP430F149 controller module, and network communication module ESP8266WIFI [33]. Among them, MSP430F149 serves as the core component. It manages the lower-level sensors and relays, facilitating data collection and appliance control. The collected data are uploaded using the computer network protocol and stored in the middle-tier Alibaba Cloud server through the MSP430F149 single-chip microcomputer and ESP8266WIFI, facilitating future user access. The MSP430F149 controller module must receive the command information from the cloud server, analyze the command content, and control the relay to make corresponding operations. The underlying architecture centered on the home gateway is a centralized network model prone to user data leakage. Smart contracts and private blockchains are employed to ensure data integrity and security to protect users’ sensitive data. This work particularly emphasizes the potential performance impacts that may exist at different stages of the system. For example, the data upload phase and cloud server processing phase may have a significant impact on overall performance. During the data upload phase, network communication and data transmission speed may be performance bottlenecks, especially when large-scale users upload data simultaneously. During the cloud server processing phase, the computing power and storage speed of the server may also have a significant impact on the system response time. Besides, an encryption algorithm is employed on the node to ensure data privacy during transmission.

### 3.1 Merkle tree

**Fig. 2:**
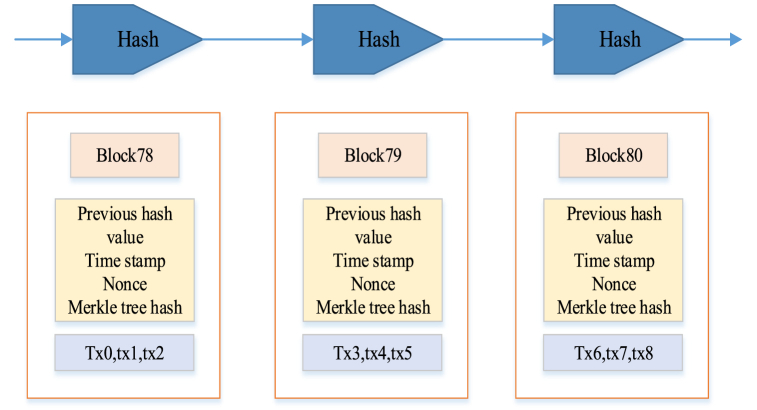
Three consecutive blocks.

Blockchain is a data structure based on time stamps. Each block contains the name and body of a block, as shown in Fig. 2.

**Fig. 3:**
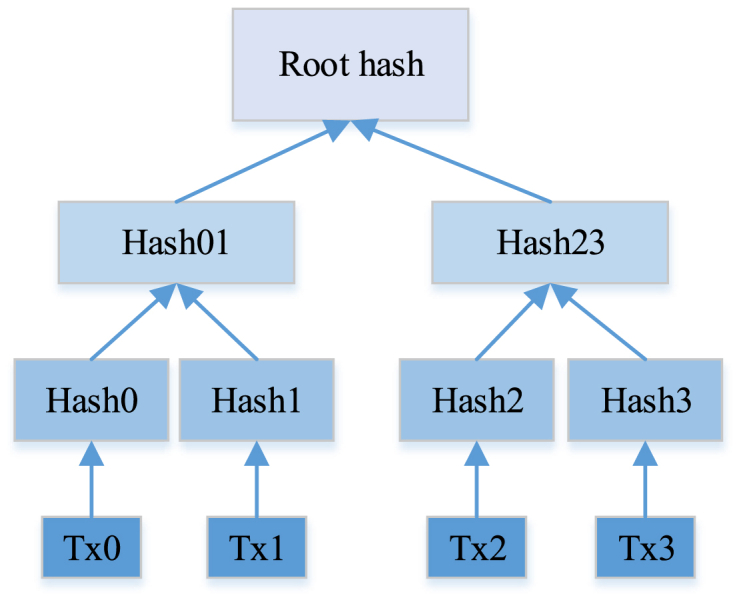
Generation of Merkle tree.

In Fig. 2, each block contains multiple transaction records, and each record has its own hash value. Additionally, each block includes the previous block's hash value to ensure the immutable sequence of blocks. The Merkle tree is a binary recursive method used to generate the root hash value of the Merkle tree. The generation of hash values involves calculations using a hash algorithm, and each hash value is derived from the previous hash value. Therefore, each block's hash value contains the previous block's hash value. This data structure design enables blockchain to achieve decentralized data storage and transaction processing. The Merkle tree root hash can be employed for confirmation when all transactions are in one block, as shown in Fig. 3.

The Merkle tree root hash is derived by recursively combining the hash values of each transaction contained in the block in a binary fashion until a single hash value occurs. Fig. 3 visually outlines the step-by-step calculation of this hash value. If any transaction belongs to a block, the Merkle tree root hash serves as a means of verification. If the existing transaction is tampered with, the hash value at the root of the Merkle tree must differ from the original hash. Consequently, the Merkle tree structure provides integrity and immutability to the blockchain.

This work delves into the security of data storage, with Merkle Tree being a key concept. In addition, to comprehensively discuss the data structures in blockchain, the concept of Merkle Patricia Tree (MPT) is introduced. MPT is an improved data structure of Merkle Tree, particularly suitable for blockchain systems. Compared to traditional Merkle trees, MPT adopts some of the ideas of Patricia trees and has been modified to meet the special needs of blockchain. The main advantage of MPT is their efficient storage and retrieval of data. MPT adopts a strategy of path compression and prefix sharing, reducing the storage of redundant data and improving the efficiency of verification [34]. The structure of an MPT can be divided into four types of nodes: extension nodes, leaf nodes, empty nodes, and branch nodes. Extension nodes are used to point to child nodes and are branches of the MPT tree. It contains a branch path that reduces storage space through prefix sharing. Leaf nodes contain actual numerical information and are the place where the MPT tree stores data. Each leaf node corresponds to a keyword and corresponding numerical value. An empty node represents an empty value or path used to maintain the structure of a tree. A branch node is one of the node types in an MPT tree, used to connect multiple nodes. MPT stores data in the form of prefix trees, and compared to traditional Merkle Tree, it can better support dynamic data insertion and deletion operations. For example, considering a blockchain that includes transaction records, in MPT, each transaction can be uniquely identified by its key information without the need to traverse the entire data structure. This design makes MPT an ideal choice for handling large amounts of data in blockchain, while maintaining efficiency and security.

### 3.2 Remote procedure call (RPC)

RPC is a distributed function interface or method interface called by users [35]. Users do not need to know the underlying problems of network technology, but only need to request services from remote computer programs through the network. The RPC uses C/S Service mode.

**Fig. 4:**
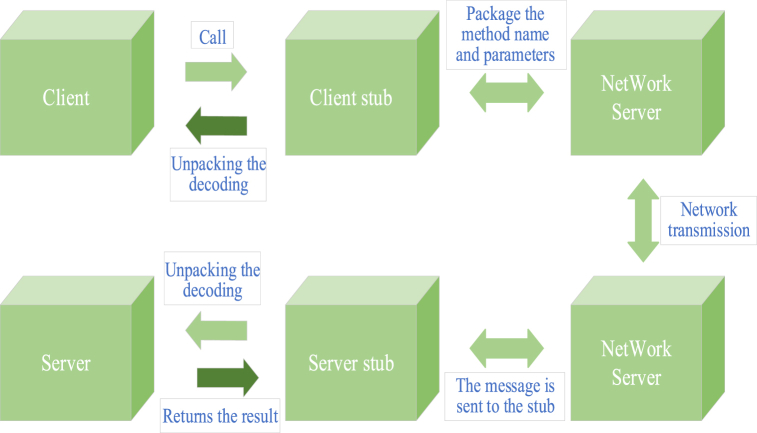
Complete RPC process.

Fig. 4 illustrates the complete process of RPC, involving the operations of different roles in RPC. Firstly, the caller initiates the call locally. This is the starting point of the RPC process, where the caller wants to execute a remote method or procedure. Secondly, the client stub intervenes and sends the message body to the server. The client stub serves as a bridge between the client and the network, responsible for passing call requests to the server. On the server side, the server stub receives messages transmitted over the network and performs unpacking and decoding operations to obtain method names and parameter information. This step is a crucial step in restoring binary data transmitted over the network into recognizable call information. Next, the server stub makes local calls based on the method name and parameters, that is, calls the actual method or procedure. The Callee performs the corresponding operation and returns the result to the server stub. The server stub is then responsible for encapsulating and encoding the returned result, forming a new message, and sending it back to the client through the network. After receiving the message returned by the server, the client stub unpacks and decodes it to restore the final call result. Finally, the client obtained the complete RPC result. The various steps in this RPC process work together organically to ensure the smooth execution of remote calls. The entire process involves multiple participating roles such as the caller, client stub, server stub, called party, and client. Information exchange is carried out through the network, achieving remote process calling and result transmission.

### 3.3 REST

REST is a kind of software architecture of client/server mode, initially proposed by Dr. Roy in a doctoral thesis published in the early 20th century. It operates in a 'stateless' manner, where client and server requests are independent, and neither retains the program state [36]. Every client request must encapsulate all the necessary information for the server to fully comprehend and execute the client's directives.

**Fig. 5:**
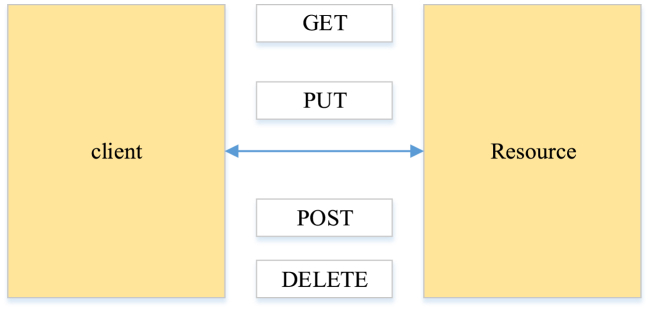
HTTP resource call.

RESTfuI Web server is a resource-oriented reading mode, prioritizing interaction with stable resources rather than focusing solely on information and behavior [37]. RESTfuI API (REST style of API): URL is adopted to find resources, HTTP verbs define actions, and response status codes signify the outcomes of operations. Fig. 5 presents the call of the REST resources: querying resources (GET), creating resources (PUT), updating resources (POST), and deleting resources (DELETE).

HTTP resource invocation is the mutual circulation between clients and resources, encompassing GET, PUT, POST, and DELETE. The client's request for a resource is processed, and the corresponding response is simultaneously relayed back to the client. A web service can be described as RESTful when it adheres to the following conditions.

1) All resources are identified and implemented by a Uniform Resource Identifier.
2) Data interaction between resources is facilitated through hyperlinks.
3) Resource operations are standardized through a uniform method.
4) Various expressions can be used between resources.
5) The connection mode between resources is allowed to be stateless. It means that all state information resides on the server side in the context of the internet. If a user wants to operate the server, specific measures must be undertaken to facilitate the server to perform state transitions. In the current market, engineers primarily rely on GET, POST, PUT, and DELETE in the HTTP protocol to achieve this.

### 3.4 Module function

**Fig. 6:**
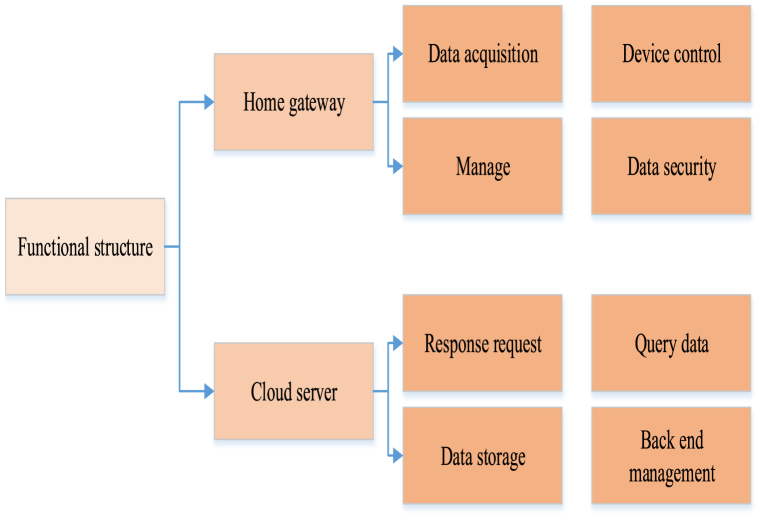
Functional structure diagram.

In the system research, redundant user functionalities have been streamlined by incorporating certain existing systems in the market. The system is divided into the home gateway and cloud service, as depicted in Fig. 6.

In Fig. 6, the home gateway module collects sensor data from the home environment and oversees the home's relay devices. It communicates with the cloud service. The cloud service module is responsible for processing data and commands and providing functionalities such as remote control and data storage. These two modules are connected and communicate with each other via the internet, forming the entire smart home system.

### 3.5 Structural interaction design

**Fig. 7:**
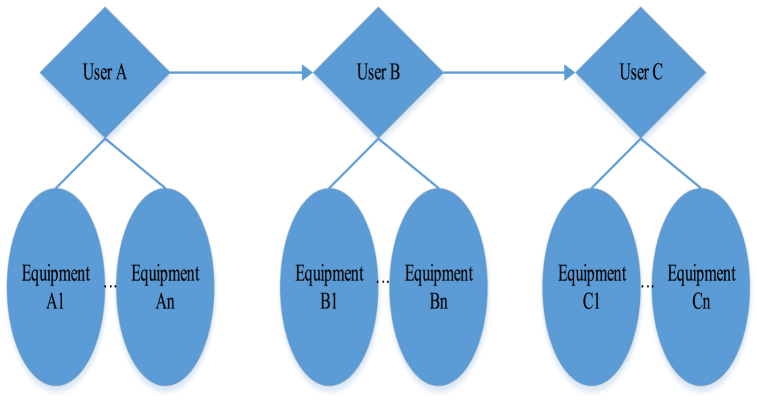
A smart home system based on BT.

In this system, human interaction has shifted from the traditional visual interaction to the interaction between people and equipment, and between equipment and equipment. This transformation necessitates the design of various nodes to achieve different interactions. Fig. 7 depicts a blockchain-based smart home architecture. Different nodes in Fig. 7 play key roles, achieving multi-level interaction. User nodes interact with the entire system through smart devices. Users can communicate with smart home systems through mobile applications or other interfaces, such as adjusting temperature, controlling lighting, etc. The device node uses intelligent devices as nodes, which can receive user instructions and perform corresponding tasks. These devices may include smart lighting fixtures and smart home appliances. Blockchain nodes use blockchain as a distributed ledger technology, responsible for recording and verifying interaction processes. Each interaction is recorded in an immutable block, ensuring system transparency and traceability. The system interaction process includes users initiating adjustment requests for smart lighting through mobile applications. After receiving the request, the device node performs corresponding operations, such as adjusting the brightness of the light. Blockchain nodes record this user device interaction, including user requests and device responses. The decentralization and encryption features of blockchain technology in blockchain-based smart home architecture ensure interaction security and prevent data tampering. The distributed ledger of blockchain ensures the transparency of the entire system, and any interaction can be verified and audited. Due to each interaction being recorded on the blockchain, the system has strong traceability, facilitating troubleshooting and historical queries. This blockchain-based smart home system achieves a safer, more transparent, and controllable smart home experience through the collaborative effect between different nodes.

### 3.6 User node interaction

**Fig. 8:**
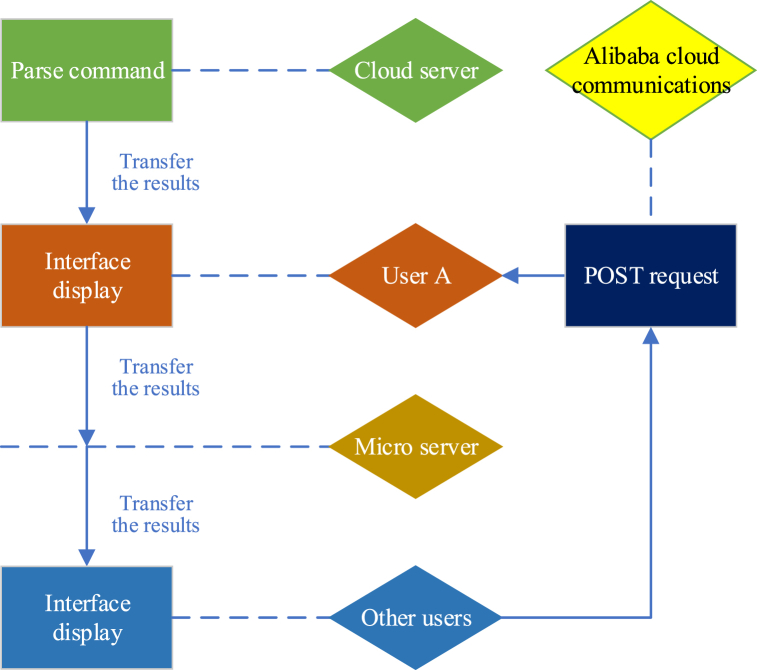
User node interaction.

This section delves into user interactions within the research system. The terminals cannot allow operations by individuals outside the authorized group to control the smart home system. Therefore, when the administrator intends to grant multiple users access to manage the same smart home system, they must input the essential information of these users into the web-based backend management system, provided they belong to the same group. Once authorized, users can collaboratively oversee the smart home system alongside others. Fig. 8 illustrates further details.

From Fig. 8, user A uses a POST request to communicate with Alibaba Cloud to realize the communication between the terminal and the cloud [38]. The cloud server can interpret the control commands from the POST request and write them into the data table. Once the cloud server has parsed the commands, it returns them to user A through an HTML5 page. After receiving the data from the cloud server, user A transmits operational instructions to the microserver. The microserver then parses the instructions and displays the operation results on the smart program interface of other users within the group.

### 3.7 Data security transmission scheme and homomorphic encryption algorithm WORKFLOW in smart home systems

**Fig. 9:**
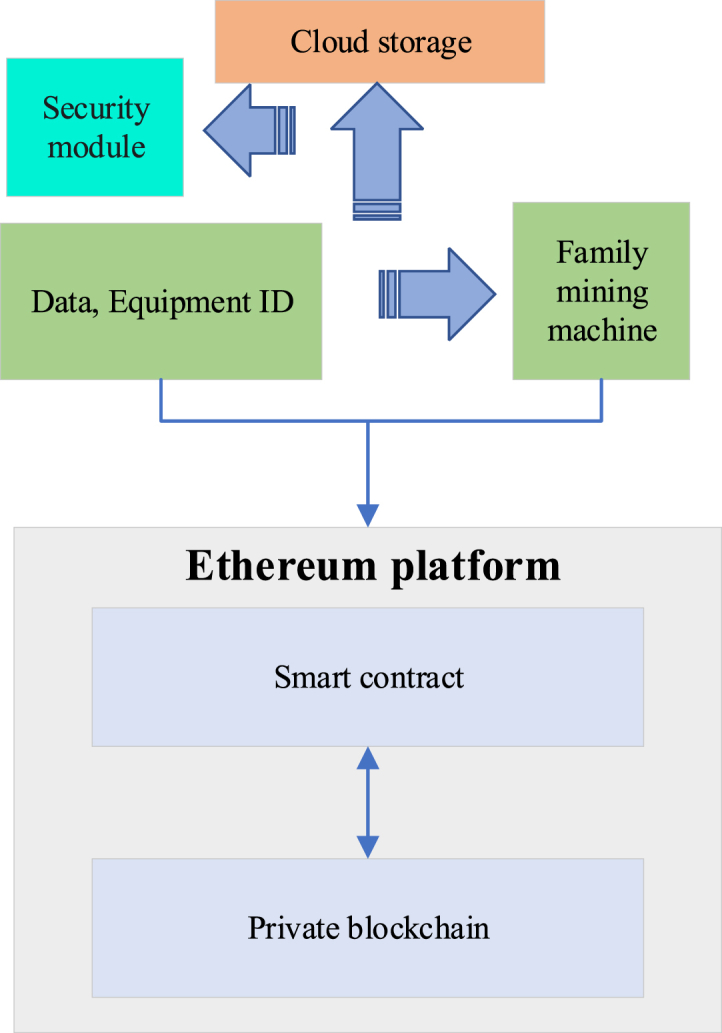
System structure of data security transmission scheme.

Fig. 9 displays the data security transfer scheme in the smart home system, including the SH microcomputer, private blockchain and cloud storage.

Household mining machines are responsible for private blockchains, which are used for data stream storage and transaction management. When using a homomorphic encryption algorithm, data detected by sensors and devices must be encrypted before being uploaded to household mining machines, thereby preventing potential interception by intermediaries [39]. The homomorphic encryption algorithm is adopted to encrypt sensor and device nodes, and upload them to MSP430F149. Additionally, a security module is integrated, encompassing functions such as data encryption and decryption, key management, and access control.

The homomorphic cryptographic algorithm can be simply summarized as follows. It is to add and multiply data of the same state and type, then encrypt it according to specific rules, then encrypt it with data, and then add and multiply. The final result remains the same. Its basic principles are as follows [40]:

If plaintext E is encrypted as f (E), and plaintext F is encrypted as f (F), f (E) and f (F) are added to obtain f (G). In ordinary cryptographic algorithms, f (G) decryption does not yield G. However, G can be derived in homomorphic ciphers by decrypting f (G), G = E + F, thus forming additive homomorphism. If the ciphertext function adheres to f (E*F) = f (E) *f (F), it is referred to as multiplicative homomorphism. When a cryptographic function can satisfy both additive homomorphism and multiplicative homomorphism, it can be called a fully holomorphic cipher. After the cipher, it can perform various encryption operations (addition, subtraction, multiplication and division, polynomial evaluation, exponent, logarithm, and trigonometric function). The system can effectively prevent users' sensitive information from being browsed to ensure users' personal information security.

### 3.8 Network security protection

The smart home system incorporates additional network security measures such as encrypted transmission, identity authentication, and firewalls. Encryption technology is employed to safeguard data during transmission, preventing data theft or tampering. User authentication is performed to ensure that only authorized users gain access, thus preventing unauthorized entry. Firewalls are set up to restrict network access to the smart home system, allowing only specific IP addresses or port numbers to access. Additionally, access by robots needs to be considered. Besides authentication mechanisms, fine-grained access control should be implemented for robot access, such as setting read-only permissions or limiting access to specific devices. Regular monitoring and logging of robot access is essential to detect any abnormal behavior and promptly take appropriate actions.

The smart home system can provide password recovery functionality in case of password forgetting. Users can verify their identity through methods like e-mail or mobile verification to retrieve their password. Regular data backups can be performed to prevent data loss or damage and facilitate data recovery. Alternatively, human customer support is available to allow users to seek assistance from customer service representatives to recover their passwords or access the system.

## 4 Experimental design and performance evaluation

### 4.1 Settings of access control algorithm

This section briefly introduces the proposed access control plan. This scheme includes six algorithms: system initialization algorithm setting, participant registration algorithm UsrReg, access control request algorithm ConReq, policy evaluation algorithm Evaluate, data acquisition algorithm DataAcq, and dispute resolution algorithm HandleDispute.

(1) System initialization serves as a security parameter that combines the system public parameter PP, the public and private keys of the group (pkG, skG), and the public and private keys of the home gateway (pkHG, skHG).
(2) Participant registration algorithm UsrReg (ID, pkHG): the participant's identification information Idi and PkHG are taken as the input of the algorithm. The algorithm outputs the participant's public-private key pair (Di, di), the participant's second anonymous identifier IDi 2, and part of the participant's private key sk.
(3) Access control requirements algorithm ConReq (Req, pkHG, di): it is taken as the PkHG of access control requirements Req.PkHG; it also includes the private key di that accesses the request. The algorithm outputs in the form of access control request Req (R, S).
(4) Policy evaluation algorithm Evaluate (R, S, H, pkG): this algorithm takes the access control request Req (R, S), access control policy H, and public key pkG as inputs, and outputs the access control decision.
(5) Data acquisition algorithm DataAcq (R, S, skG, H): this algorithm takes the access control request Req (R, S), group private key skG, and access control policy H as inputs, and outputs the requested data for the user.
(6) Dispute resolution algorithm HandleDispute (Req, R′, S′, sk, skG): this algorithm takes the access control request Req (R, S), access control decision Req (R′, S′), user's private key sk, and group private key skG as inputs, and outputs the dispute resolution result.

System latency typically refers to the time elapsed between data input and output. The computational expression reads:

(1) $$Delay=\frac{sum\left\{{Res}_{all}\right\}}{\left\{{Req}_{tot}\right\}}$$

sum represents the summation symbol, ${Res}_{all}$ stands for the response time for all requests, and ${Req}_{tot}$ represents the total number of requests. Throughput typically signifies the number of transactions or requests the system processes within a specified time frame. The computational expression reads:

(2) $$Throughput=\frac{\left\{{Tran}_{c}\right\}}{\left\{T\right\}}$$

${Tran}_{c}$ denotes the number of completed transactions, and T represents the total time.

### 4.2 Experimental environment

It is assumed that there are three different organizations: OrgE, OrgF and OrgG. Moreover, all smart home data resource providers are members of OrgE, and each resource provider has a Peer node running under OrgE. OrgF is an organization that represents all access requests, with five Peer nodes below it. Finally, it is assumed that OrgG represents all regulatory agencies, with three operational peers under it. The peers of OrgE in the operating environment are Intel (R) 3.6 GHz, 16G running memory, and Intel (R) Corei 7-7700 of Ubuntul6.04. The peer nodes of OrgE and OrgF run in the same configuration as OrgE. In the classification service, two Order nodes are provided by the kafka-zookeeper cluster. The working environment of Order is the same as that of OrgE.

The sensors are divided into three groups on the smart home network lab bench. Each group is equipped with four FIWARE devices. FIWARE devices are a set of hardware and software components designed to facilitate the development and deployment of IoT applications. These devices provide a standardized interface for communication with sensors and other IoT devices. This work utilizes FIWARE devices as part of the smart home network experimental platform. Each group of sensors is connected to four FIWARE devices. Among them, three are used as smart home terminal devices, and one is used as a router. Each group runs a separate gateway program. The gateway program is written in Java. My SQL database is applied to store routing tables, resource catalogs, and data tables. Each group and the gateway form a separate smart home network. Device discovery and resource discovery in the smart home network are performed according to the IEEE 802.15.4 standard.

### 4.3 Parameters setting

This work uses the FabricClientSDK to write a client application software based on Java [41]. This client application offers a user-friendly interface for attribute creation, assigning attributes to specific entities, and creating Attribute-Based Access Control (ABAC) policies for specific resources.

The ABAC algorithm is an attribute-based access control algorithm that establishes an attribute mapping relationship between access requests and authorization decisions. Specifically, in this algorithm, access requests and authorization decisions can be matched based on multiple attributes (like user identity, roles, organization, and time) to determine if a user has the privilege to access specific resources. In the start-up phase, the MSP of each institution provides participants with encryption components such as certificates, signing keys, and encryption keys. Subsequently, distinct support strategies are devised for the three smart home groups, as detailed in Table 2.

**Table 2:** Endorsement strategies for data access control of different smart home groups.

ID	1	2	3
Endorsement strategy	Any peer node	There is at least one peer node.	All peer nodes.
Smart home group	Group 1	Group 2	Group 3

Following the determination of the support policy, three smart contract algorithms are installed on each Peer node: attribute management AttributeMgr, policy management PolicyMrg, and access control decision ACDecMaker. On this basis, the MSP of each institution establishes the attributes of subjects and objects on the blockchain. In orgC, each institution's MSP establishes the environment and license attributes. Finally, the client application creates the target attribute of each smart home group [42]. During the attribute creation process, these attributes are assigned to different objects. On this basis, 20 target attributes, 20 object attributes, 10 environment attributes and 5 permission attributes are established. Five different ABAC strategies are created, and the required attributes range from 10 to 50.

### 4.4 Performance evaluation

**Fig. 10:**
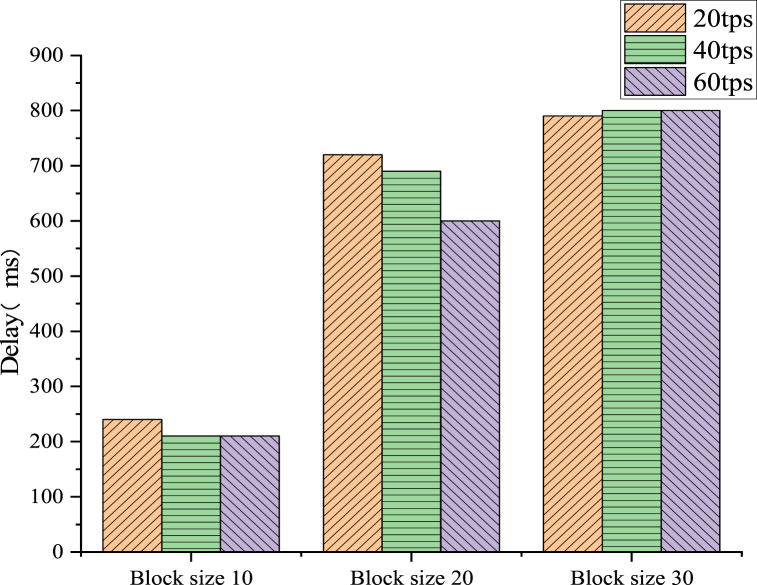
Property creation transaction delay.

**Fig. 11:**
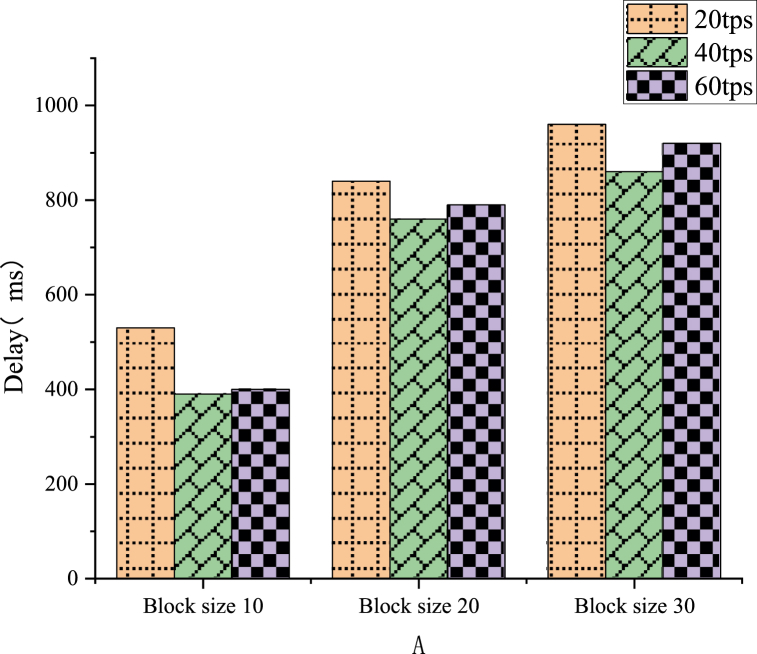
Property assignment transaction delay.

The execution speed of the ABAC strategy determines the performance of this scheme. The average of these results is obtained through five experiments. The three main configurable parameters are stateDB, endorsement strategy, and block size [43]. In Goleve1DB and CouchDB, the existing literature reveals that Goleve1DB exhibits superior processing power and higher reading and writing speed when the data are small. The last written data block has reached a block size, or the amount of suspended data will increase by a new data block after the timeout. In this test, the timeout is set to 1 s. Then, under three different transaction arrival rates, the actions of creating attributes and assigning attributes at the 10, 20 and 30 block sizes are executed, respectively. Fig. 10, Fig. 11 demonstrate that the delay increases as the block size grows. The tps in the figure represents transactions per second, a metric for assessing the performance of a blockchain network. It indicates the number of transactions processed per second in a blockchain network.

In Fig. 10, the abscissa is the different block size values. The vertical axis is the transaction delay time. Different legends represent different transaction arrival rates. The data results demonstrate that the latency increases as the block size grows. For instance, increasing the block size from 10 to 30 results in a threefold increase in delay, from 215 ms (ms) when the arrival rate of attribute creation is 40. This is because as the block size expands, pending transactions must wait longer in the message queue, which can cause transaction rate delays in the BT.

The experimental results in Fig. 11 suggest that between attribute creation and attribute assignment, attribute assignment takes a long time on average. This extended duration can be attributed to the Attribute Mgr smart contract algorithm within the scheme, which necessitates the verification of signature values in the X.509 attribute certificate. This is an expensive cryptographic operation. Overall, the system exhibits the lowest latency when the transaction arrival rate is 40tps and the block size is 10.

A set of delays related to smart home network resource access without access control strategy is designed and compared with three different smart home groups established to compare with normal conditions. Table 3 reveals the results.

**Table 3:** Smart home resource access request delay.

Number of attributes	Group 1 (ms)	Group 2 (ms)	Group 3 (ms)	No access control policy (ms)
10	0.7	1.5	2.9	0.4
20	0.7	1.7	3.2	0.4
30	1.0	1.8	3.3	0.4
40	1.4	1.9	3.4	0.4
50	1.5	2.1	3.9	0.4

Table 3 shows that the lowest delay occurs in the absence of access control policies. Furthermore, Group 3 exhibits a higher latency than Groups 1 and 2. The reason is that the endorsement policy of Group 3 requires 11 peer nodes to endorse a transaction, while Groups 1 and 2 only need 1 and 3 peer nodes, respectively, for endorsement. The scheme based on the Hyperledger Fabric consortium chain proposed here can serve smart home resource access requests faster than public blockchains. Additionally, the greater the number of validating peer nodes in the endorsement policy is, the higher the system's security level is and the greater the communication and computational overhead is. With proper security parameter settings, a trade-off between security and practicality can be achieved in smart home access control systems. The simulation results underscore that the access control scheme proposed here meets the actual application requirements within the appropriate security parameter range. Table 4 provides a performance comparison of different methods for smart home access control systems. It demonstrates that the consortium chain based on Hyperledger Fabric exhibits the lowest system latency (0.4 ms) and the highest throughput (45tps). In contrast, the IoT platform-based approach exhibits relatively higher system latency (1.2 ms) and lower throughput (30tps). The traditional database-based method shows moderate system latency (1.5 ms) and lower throughput (25tps). The Ethereum-based smart contract approach exhibits lower system latency (0.9 ms) and higher throughput (35tps). This indicates that this system offers fast response times and robust transaction processing capabilities, although it is slightly lower compared to the Hyperledger Fabric-based method. In summary, the smart home access control system based on the Hyperledger Fabric consortium chain demonstrates superior performance in terms of system latency and throughput, followed by the Ethereum-based approach, while the methods based on the IoT platform and traditional database exhibit comparatively lower performance.

**Table 4:** Performance Comparison of different methods for Smart Home Access Control Systems.

Methods	System latency (ms)	Throughput (tps)
Based on the Hyperledger Fabric consortium chain	0.4	45
Based on the IoT platform	1.2	30
Based on traditional database	1.5	25
Based on Ethereum smart contracts	0.9	35

### 4.5 DISCUSSION

The smart home access control system based on the public link still suffers from high transaction delays [44]. For instance, processing a Bitcoin transaction through the blockchain may take several hours. Therefore, this scheme is unsuitable for resource access control in all smart home networks because they require low transaction delay. For example, emergency services require quick access to patients' wearable devices. The experimental results on the test platform demonstrate that this method can enhance the access control performance of the blockchain network. The optimal parameters are 20 blocks, 40 transactions per second, and the delay of accessing data resources is about 1 s. In this way, the best parameter value can be used to make the access control system complete the access to home resources faster.

Compared with the existing BT, the proposed scheme based on the Hyperledger Fabric consortium chain can provide faster access to family resources. Additionally, it's important to note that as the number of validating nodes in the endorsement policy increases, system security rises, but communication and computational overhead also escalates. The smart home access control system can achieve the right balance between security and practicality through well-configured security parameters. The simulation results suggest that the designed access control system can effectively meet real-world requirements under appropriate security settings.

Therefore, technology research and development in the field of smart homes will be a focus in the future [45]. In order to build a robust smart home interconnection system and deliver valuable services to users, sharing smart home data with the outside world is often necessary. However, these shared data often contain substantial sensitive and private user information. Currently, there is a notable absence of an effective access control scheme for smart home data to ensure user data security, and this presents a bottleneck in the advancement of smart homes [46]. Consequently, many challenges remain to be addressed in smart home data access control, with only a fraction of these issues currently resolved due to limited knowledge, capabilities, and expertise.

This work investigates a blockchain-based access control system for smart homes, with a particular focus on identity-based proxy aggregation signature schemes. Unlike the study by Lin et al. (2019) [47], this work mainly focuses on the application of blockchain in the security and performance of smart homes. Dang's research focuses on smart home systems based on BT, emphasizing the performance of SHIB systems in data privacy, trust access control, and scalability. In contrast, this work explores how blockchain technology can improve the security of data storage and connections by introducing identity-based proxy aggregation signature schemes to improve efficiency and reduce the pressure on storage space and communication bandwidth. Compared with the study by Zhang et al. (2021) [48], this work not only focuses on the privacy and integrity of blockchain in smart homes, but also specifically addresses the issues of limited transaction storage space and slow speed in blockchain transactions. To address these issues, this work proposes an IBPAS scheme that utilizes identity-based proxy aggregation signature schemes to improve the efficiency of signature verification, compress storage space, and reduce communication bandwidth. Compared with the work of Wu et al. (2023) [49], this work emphasizes the key factors in improving performance in smart home access control systems. The identity-based proxy aggregation signature scheme proposed by Wu et al. aims to improve the efficiency of signature verification, while this work demonstrates the effectiveness of this scheme in practical applications through experiments and performance evaluations of the system. In addition, this work also considers the impact of access control policies on performance, providing a comprehensive understanding of system performance through a detailed analysis of latency and throughput. In terms of specific technical details, the identity-based proxy aggregation signature scheme proposed here has been practically applied. The experimental results show that as the block size increases, transaction latency shows an increasing trend. This is consistent with the actual operation of the system, as an increase in block size may lead to an increase in the waiting time for pending transactions in the message queue, thereby causing a delay in the transaction rate in BT. Moreover, a detailed analysis is conducted on the latency of accessing smart home resources, and in the Hyperledger Fabric consortium chain, the system shows the lowest latency and highest throughput, with better performance compared to other methods. Overall, the research findings of this work provide in-depth insights into the performance advantages and key factors of blockchain-based smart home access control systems. By comparing with the work of relevant scholars, the superiority of the proposed solution in improving efficiency, reducing storage pressure, and increasing communication bandwidth is emphasized. This work has positive practical significance for promoting the development of smart home systems, enhancing their safety and performance.

## 5 CONCLUSION

### 5.1 RESEARCH conclusion

In order to study the design of BT-based smart home systems, this work analyzes the architecture of smart home systems, delves into the functions and designs of two main modules, namely home gateway and cloud services, and proposes a data security transmission solution for smart home systems. The simulation experiments yield the following conclusions. (1) The delay time for smart home resource access requests increases with the increase of block size, ranging from a minimum delay of 210 ms to a maximum delay of 800 ms; (2) The system exhibits the lowest delay when the transaction arrival rate is 40tps and the block size is 10; (3) In the comparative experiment of delay for smart home network resource access, the access control solution based on Hyperledger Fabric consortium chain outperforms other methods. It balances security and practicality within an appropriate range of security parameters.

### 5.2 FUTURE WORKS and research limitations

As digital science and technology continue to advance, multiple applications and services in the smart home field have attracted more attention. This work designs and implements a smart home system, and selects sensors and relays to form the home system's core part for monitoring and controlling the home environment.

#### 5.2.1 RESEARCH deficiencies

First, this work delves into smart home and data security, particularly discussing smart home access based on BT. The findings highlight that smart home access predominantly revolves around mobile terminals, which are susceptible to external attacks due to network distribution and resource limitations.

The proposed BT-based smart home access control scheme uses attribute-based access control as the access control model. The access request of smart home data resources is satisfied by designing a consensus algorithm based on BT. Therefore, future work should aim to reduce latency by optimizing this algorithm. Furthermore, the proposed scheme has only been implemented on the simulation platform and is not deployed in the actual hardware environment or application scenarios. Therefore, further research should focus on practical applications to enhance the system's real-world usability.

#### 5.2.2 FUTURE research

In order to address the security problems in data storage, this work proposes a distributed key storage method based on a verification equation, improving a smart home's security.

The proposed BT-based smart home control system addresses existing residential access control problems. This method can ensure that resource participants can participate in the whole access control process to avoid the risk of permission access caused by the centralized management of the third party. However, with the advent of 5G technology, the combination of new-generation mobile communication technology and various industries, and the rapid advancements of software and hardware technology, the smart home system remains a hot topic with promising prospects for future development.

## CRediT authorship contribution statement

**Ying Huang:** Writing – review & editing, Writing – original draft, Software, Methodology, Formal analysis, Data curation, Conceptualization.

## Declaration of competing interest

The authors declare that they have no known competing financial interests or personal relationships that could have appeared to influence the work reported in this paper.

## References

[1] Jumriani J. (2021). The urgency of local wisdom content in social studies learning: literature review. The Innovation of Social Studies Journal.

[2] Kim H. (Apr. 2021). A systematic review of the smart energy conservation system: from smart homes to sustainable smart cities. Renew. Sustain. Energy Rev..

[3] Sepasgozar S., Karimi R., Farahzadi L., Moezzi F., Shirowzhan S., Ebrahimzadeh S.M., Hui F., Aye L. (Apr. 2020). A systematic content review of artificial intelligence and the internet of things applications in smart home. Appl. Sci..

[4] Machorro-Cano I., Alor-Hernández G., Paredes-Valverde M.A., Rodríguez-Mazahua L., Sánchez-Cervantes J.L., Olmedo-Aguirre J.O. (Oct. 2020). HEMS-IoT: a big data and machine learning-based smart home system for energy saving. Energies.

[5] Zhang X., Zhang Z. (Dec. 2020). How do smart villages become a way to achieve sustainable development in rural areas? Smart village planning and practices in China. Sustainability.

[6] Saleem M.U., Shakir M., Usman M.R., Bajwa M.H.T., Shabbir N., Shams Ghahfarokhi P., Daniel K. (Jun. 2023). Integrating smart energy management system with internet of things and cloud computing for efficient demand side management in smart grids. Energies.

[7] Mallinson D.J., Shafi S. (Feb. 2022). Smart home technology: challenges and opportunities for collaborative governance and policy research. Rev. Pol. Res..

[8] Haque A.B., Bhushan B., Dhiman G. (Jun. 2022). Conceptualizing smart city applications: requirements, architecture, security issues, and emerging trends. Expet Syst..

[9] Singh S., Sh-BlockCC (2019). A secure and efficient Internet of things smart home architecture based on cloud computing and blockchain technology.”. Int. J. Distributed Sens. Netw..

[10] Lee Y. (Mar. 2020). A blockchain-based smart home gateway architecture for preventing data forgery. Hum-cent Comput Info.

[11] Mansouri S.A. (Jun. 2021). Energy management in microgrids including smart homes: a multi-objective approach. Sustain. Cities Soc..

[12] Zang M. (Dec. 2019). Blockchain-enabled decentralized trust management and secure usage control of IoT big data. IEEE INTERNET THINGS.

[13] Zheng K. (Dec. 2022). Blockchain technology for enterprise credit information sharing in supply chain finance. J INNOV KNOWL.

[14] Dang T.L.N., Nguyen M.S. (2018).

[15] Tchagna Kouanou A. (Feb. 2022). Securing data in an internet of things network using blockchain technology: smart home case. SN Computer Science.

[16] Khan M.A. (Nov. 2020). A machine learning approach for blockchain-based smart home networks security. IEEE Network.

[17] Shahbazi Z., Byun Y.C., Kwak H.Y. (Sep. 2021). Smart home gateway based on integration of deep Reinforcement learning and blockchain framework. Processes.

[18] Singh P.K. (2019). International Conference on Innovations for Community Services.

[19] Ren Y. (Feb. 2021). Multiple cloud storage mechanism based on blockchain in smart homes. Future Gener Comput Syst.

[20] Ammi M., Alarabi S., Benkhelifa E. (May. 2021). Customized blockchain-based architecture for secure smart home for lightweight IoT. INFORM PROCESS MANAG.

[21] Farooq M.S., Khan S., Rehman A., Abbas S., Khan M.A., Hwang S.O. (Jun. 2022). Blockchain-based smart home networks security empowered with fused machine learning. Sensors.

[22] Menon S., Anand D., Kavita, Verma S., Kaur M., Jhanjhi N., Ghoniem R.M., Ray S.K. (Jul. 2023). Blockchain and machine learning Inspired secure smart home communication network. Sensors.

[23] Liao Z., Pang X., Zhang J., Xiong B., Wang J. (Oct. 2021). Blockchain on security and forensics management in edge computing for IoT: a comprehensive survey. IEEE Transactions on Network and Service Management.

[24] Wang J., Wei B., Zhang J., Yu X., Sharma P.K. (Aug. 2021). An optimized transaction verification method for trustworthy blockchain-enabled IIoT. Ad Hoc Netw..

[25] Dhanaraj R.K., Kadry S., Kang B.-G., Nam Y. (Nov. 2022). Probit cryptographic blockchain for secure data transmission in intelligent transportation systems. J. Internet Technol..

[26] Zhang J., Zhong S., Wang T., Chao H.-C., Wang J. (Jan. 2020). Blockchain-based systems and applications: a survey. J. Internet Technol..

[27] Wang J., Chen W., Wang L., Sherratt R.S., Alfarraj O., Tolba A. (Jul. 2020). Data secure storage mechanism of sensor networks based on blockchain. Comput. Mater. Continua (CMC).

[28] Singh S.K., Azzaoui A., Kim T.W., Pan Y., Park J.H. (Mar. 2021). DeepBlockScheme: a deep learning-based blockchain driven scheme for secure smart city. Human-centric Computing and Information Sciences.

[29] Ren Y., Leng Y., Qi J., Sharma P.K., Wang J., Almakhadmeh Z., Tolba A. (Feb. 2021). Multiple cloud storage mechanism based on blockchain in smart homes. Future Generat. Comput. Syst..

[30] Ren Y., Leng Y., Cheng Y., Wang J. (Mar. 2019). Secure data storage based on blockchain and coding in edge computing. Math. Biosci. Eng..

[31] Mukta M.Y. (May. 2020). IoT for energy efficient green highway lighting systems: challenges and issues. J. Netw. Comput. Appl..

[32] Apoorva K.A., Sangeetha S. (Jun. 2022). Analysis of uniform resource locator using boosting algorithms for forensic purpose. Comput. Commun..

[33] Chung J.-j., Kim H.-J. (Mar. 2020). An automobile environment detection system based on deep neural network and its implementation using IoT-enabled in-vehicle air quality sensors. Sustainability.

[34] Heshmati A., Bayat M., Doostari M., Pournaghi S.M. (Dec. 2023). Blockchain based authentication and access verfication scheme in smart home. J. Ambient Intell. Hum. Comput..

[35] Hatledal L.I. (Aug. 2019). A language and platform independent co-simulation framework based on the functional mock-up interface. IEEE Access.

[36] Wu B., Chen T., Yang K., Wang X. (Mar. 2021). Edge-centric bandit learning for task-offloading allocations in multi-RAT heterogeneous networks. IEEE Trans. Veh. Technol..

[37] Panayiotou K. (Mar. 2022). A framework for rapid robotic application development for citizen developers. Software.

[38] Xu Y., Gui G., Gacanin H., Adachi F. (Feb. 2021). A survey on resource allocation for 5G heterogeneous networks: current research, future trends, and challenges. IEEE Communications Surveys & Tutorials.

[39] Shrestha R., Nam S.Y., Bajracharya R., Kim S. (Aug. 2020). Evolution of V2X communication and integration of blockchain for security enhancements. Electronics.

[40] Catak F.O. (Jan. 2020). Practical implementation of privacy preserving clustering methods using a partially homomorphic encryption algorithm. Electronics.

[41] Islam M.A., Madria S. (Jul. 2019). Blockchain.

[42] Qashlan A. (Jul. 2021). Privacy-preserving mechanism in smart home using blockchain. IEEE Access.

[43] Gupta M. (Sep. 2020). An attribute-based access control for cloud enabled industrial smart vehicles. IEEE Trans Industr Inform.

[44] Sookhak M. (Mar. 2021). Blockchain and smart contract for access control in healthcare: a survey, issues and challenges, and open issues. J. Netw. Comput. Appl..

[45] Mocrii D., Chen Y., Musilek P. (Sep. 2018).

[46] Aledhari M. (Jul. 2020). Federated learning: a survey on enabling technologies, protocols, and applications. IEEE Access.

[47] Lin C., He D., Kumar N., Huang X., Vijayakumar P., Choo K.-K.R. (Feb. 2019). HomeChain: a blockchain-based secure mutual authentication system for smart homes. IEEE Internet Things J..

[48] Zhang J., Zhong S., Wang J., Yu X., Alfarraj O. (Nov. 2021). A storage optimization scheme for blockchain transaction databases. Comput. Syst. Sci. Eng..

[49] Wu F., Zhou B., Zhang X. (Mar. 2023). Identity-based proxy signature with message recovery over NTRU lattice. Entropy.

